# LASSO regression and WGCNA-based telomerase-associated lncRNA signaling predicts clear cell renal cell carcinoma prognosis and immunotherapy response

**DOI:** 10.18632/aging.205871

**Published:** 2024-05-30

**Authors:** Cheng Shen, Kaiyao Jiang, Wei Zhang, Baohui Su, Zhenyu Wang, Xinfeng Chen, Bing Zheng, Tao He

**Affiliations:** 1 Department of Urology, The Second Affiliated Hospital of Nantong University, Nantong, Jiangsu 226001, China; 2 Medical Research Center, The Second Affiliated Hospital of Nantong University, Nantong, Jiangsu 226001, China; 3 Party Committe and Hospital Administration Office, The Second Affiliated Hospital of Nantong University, Nantong, Jiangsu 226001, China

**Keywords:** renal clear cell carcinoma, telomerase genes (TRs), immune infiltration, signature

## Abstract

Objective: To investigate whether telomerase-associated lncRNA expression affects the prognosis and anti-tumor immunity of patients with renal clear cell carcinoma (ccRCC).

Methods: A series of analyses were performed to establish a prognostic risk model and validate its accuracy. Immune-related analyses were performed to assess further the association between immune status, tumor microenvironment, and prognostic risk models.

Results: Eight telomerase-associated lncRNAs associated with prognosis were identified and applied to establish a prognostic risk model. Overall survival was higher in the low-risk group.

Conclusion: The established prognostic risk model has a good predictive ability for the prognosis of ccRCC patients and provides a new possible therapeutic target for ccRCC.

## INTRODUCTION

Renal cell carcinoma is a frequent genitourinary cancer that claims the lives of around 170,000 people annually. Clear cell renal cell carcinoma (ccRCC), which accounts for around 80% of cases, is RCC’s most prevalent histological form [1]. Metastasis is typically already established at the time of diagnosis since renal clear cell carcinoma is asymptomatic. Recurrence after nephrectomy is frequent, and surgical excision of RCC metastases is likewise challenging. Furthermore, insensitivity to chemotherapy and radiation distinguishes ccRCC from other urologic malignancies [2]. Due to its high immunogenicity, ccRCC may respond favorably to immunotherapy. Immunotherapy has undoubtedly made significant progress in the treatment of ccRCC, although individual differences still affect treatment outcomes [3]. Clinical prognostic models have recently been developed to successfully predict recurrence-free survival time in renal cancer [4]. In addition, Jialin Meng et al. analyzed the predictive value of cell division cycle-related proteins in patients with clear cell renal cell carcinoma [5]. Because of the heterogeneity of ccRCC patients, however, we urgently need to develop accurate, comprehensive risk models, stratify patients, and design personalized treatment options in terms of prognosis prediction and drug selection.

Telomerase is the enzyme that causes telomere elongation in cells. It lengthens telomeres and increases the number of cell divisions by adding telomeric DNA to the ends of eukaryotic cells’ chromosomes and replacing telomeres lost during DNA replication [6]. Telomerase is essential to preserve genomic integrity, telomere stability, and continued proliferative potential [7]. Normal human tissues and tumors exhibit reactivated telomerase activity, which may play a role in developing malignant transformation [8]. Almost all cancers have tumorigenic properties regulated by telomeres and telomerase. These characteristics include resistance to cell death, activation of invasion and metastasis, avoidance of growth inhibitors, genomic instability, maintenance of proliferative signals, induction of angiogenesis and immune regulation, and maintenance of proliferative signals [9]. Telomere control is, therefore, crucial to the development of tumors. Furthermore, it has been demonstrated that the telomere length of tumor cells is a reliable indicator of patient survival and therapeutic response [10, 11].

Long non-coding RNAs, or lncRNAs, are RNAs longer than 200 bp that do not code for proteins. They regulate immunological responses, including immune cell infiltration, antigen recognition, antigen exposure, and tumor development [12]. By altering gene transcription and post-transcription, lncRNAs contribute to distinct aspects of carcinogenesis and metastasis [13–15]. Androgen receptors have been reported to be able to alter TWIST1 nonsense-mediated decay via lncRNA-TANAR and promote vasculogenic mimicry (VM) in renal cell carcinoma (RCC) [16]. Long non-coding lncRNAs have been demonstrated to regulate telomerase-related pathways, which is essential for the early development of cancer [17]. Nevertheless, studies on TR-associated lncRNAs’ function in tumor immunity (TIME) and the prognosis of ccRCCs are still lacking.

Therefore, to test the predictive value of the TRL model in predicting the prognosis of patients with colorectal cancer and their response to chemotherapy and immunotherapy, we carried out a thorough systematic study to identify TR-associated genes by weighted gene co-expression network analysis (WGCNA) and then establish a TRL model risk profile by LASSO regression. The findings of this study will provide fresh light on how TR affects ccRCC and contribute to improving ccRCC patients’ tailored treatments. Lastly, the *in vitro* signature lncRNA investigations prove the model’s dependability.

## MATERIALS AND METHODS

### Acquisition and processing of data

The TCGA website (https://portal.gdc.cancer.gov/) provided RNA-seq data with FPKM normalization for renal clear cell carcinoma (TCGA-KIRC) along with related clinical and prognostic data; 613 patients’ lncRNA expression and survival time data were also retrieved. In total, 507 patients who had been followed up for more than 30 days met the inclusion criteria. Patients were randomly split into two groups: a test group (*n* = 253) and a training group (*n* = 256). A complete set of 298 genes associated with TR was downloaded from the GeneCards website (https://www.genecards.org).

### Screening of differential genes

First, we eliminated all telomerase genes (TRs) having a correlation value less than 7. Following previously described procedures [18], we further preprocessed the data using the limma program at false discovery rate (FDR) < 0.05 and | log2 fold change (FC) | > 1. We then chose 210 differentially expressed TRs for additional analysis. As per the earlier explanation [19], ccRCC-related modules were found using the WGCNA package (version 1.61). In short, a scale-free topology criterion was used to compute soft thresholds. A minimum module size of thirty genes was established by selecting the best soft threshold. The dynamic tree cut recognition module was utilized to set the MEDissThres parameter to 0.25.

### Construction and validation of TR-associated lncRNA risk models

Data from CcRCC patients were randomized 1:1 to the training or test sets. A risk model for TR-associated lncRNAs was built using the training set, and the risk model was validated using the test set and the entire set. Using univariate Cox regression analysis, TR-related lncRNAs linked to the prognosis of renal carcinoma were found. Using multiple Cox regression analysis and the LASSO Cox regression technique, a prognostic risk model based on optimum lncRNAs was created. Every person was given a risk score based on this risk model. The following is the calculation of risk scores: Risk score = Σi = 1nCoef (i) × Expr (i). Where Expr (i) denotes the normalized expression level for each lncRNA and Coef (i) denotes the regression coefficient for each lncRNA. The training set was split into low and high-risk groups based on the median risk score. To find out if there were any differences in overall survival between the two risk groups, we used K-M curves. We evaluated the accuracy of the risk model using the concordance index (C-index), produced receiver operating characteristic (ROC) curves for clinical features and prognostic models, and measured the area under the curve (AUC).

### PCA, functional enrichment analysis

The geographic distribution of the two risk groups across four expression profiles—the total gene expression profile, the telomerase gene expression profile, the telomerase-associated lncRNA expression profile, and six telomerase-associated lncRNA expression profiles in the risk model—was examined using principal component analysis (PCA). With the “ggplot2” software, we carried out a genome encyclopedia (KEGG) study. We found mRNAs that were strongly correlated with the lncRNAs above to explore the biological function of these eight lncRNAs in ccRCC. Sankey diagrams created and showed a co-expression network comprising lncRNAs and mRNAs. The threshold for the correlation coefficient was established at > 0.4 or < − 0.4, and a *P*-value of less than 0.001 was deemed statistically significant.

### Construction of nomograms

To create nomogram survival maps that could predict the 1-year, 2-year, 3-year, and 5-year survival of ccRCC patients, we combined the risk score with age, sex, stage, and clinicopathological features of the M stage. We then used the calibration curve to determine whether the predicted survival rate was consistent with the actual survival rate.

### Predictive signature drug sensitivity analysis

We assessed the contribution of predictive variables in predicting ccRCC treatment response using the Cancer Drug Sensitivity Genomics (GDSC) database. This publicly available resource compiles data on cancer cell drug sensitivity and molecular indicators of drug response [20]. GDSC2 gene expression profiles and related drug response information were obtained using the oncoPredict software [21]. The half maximum inhibitory concentration (IC50) of each medication in individuals with ccRCC was predicted using sensitivity ratings.

### Immune infiltrate level analysis

Additionally, using the “gsa” software, ssGSEA was used to quantify immune cells and pathways between the two groups. The “GSVA” software package was utilized to compute the infiltration score of 16 immune cells and the activation of 13 immune-related pathways by single-sample gene set enrichment analysis (ssGSEA) [22]. Lastly, the association between the risk score and immunological checkpoints was examined by identifying variations in gene expression levels between high-risk and low-risk groups.

### Cell culture

We acquired human renal cell carcinoma cell lines (769-P, 786-O, ACHN, CAKI-1, OSRC2) and human tubular epithelial cells (HK2) from Purcell Life Technologies Co., Ltd. The cells were cultivated at 37°C with 5% CO_2_ in a similar medium (Thermo Fisher Scientific, Inc.) supplemented with 10% fetal bovine serum (FBS; Gibco).

### RNA extraction and real-time quantitative PCR (RT-qPCR) analysis

TRIzol^®^ (Qiagen, Inc.) isolated total RNA from RCC cells and tissues. Thermo-Script Reverse Transcription Kit (Thermo Fisher Scientific, Inc.) synthesized cDNA by the reagent manufacturer’s instructions. Using a CFX96™ Real-Time PCR System (Bio-Rad Laboratories, Inc.) and SYBR Green reagent (Takara Bio, Inc.), ten μl reactions were put onto 96-well plates for qPCR. 95°C for 5 minutes, 95°C for 15 seconds, 60°C for 25 seconds, 72°C for 30 seconds, and 40 cycles were the thermal cycling conditions. The 2^−ΔΔCq^ technique was employed to calculate the relative gene expression levels [23]. A regulating gene for normalizing gene expression was beta-actin. Supplementary Table 1 lists the primers used in the real-time PCR acquired from Sangon Biotech (Shanghai, China).

### Proliferation and migration assay

Twenty-four hours after transfection, the cells were evenly distributed in 96-well plates with five parallel replicate wells for each group, and ten μL CCK-8 reagent was added on days 1, 2, and 3 to determine the absorbance at 450 and 630 nm, representing the cell growth rate. Renal cell carcinoma cells (5 × 10^4^) transfected with AC002451.1 knockdown plasmid or negative control were added to the upper chamber of transwell plates containing 200 μL serum-free medium and 800 μL medium containing 10% fetal bovine serum. The lower chamber acts as an inducer. After 24 h, migrated cells were washed with PBS, fixed in methanol, and stained with 1% crystal violet, and non-migrated cells in the upper chamber were removed with a cotton swab. At least four randomly selected fields were observed and counted under a microscope (Olympus, Tokyo, Japan).

### Wound healing test

Cells were incubated in 6-well plates. When the cells were cultured to 80% confluence, scratches were performed using a 200 μL pipette tip. Cells were washed with PBS and placed in the serum-deficient medium for 24 hours. The wound healing area was recorded at 0 and 24 hours using a microscope (Olympus, Tokyo, Japan).

### Statistical analysis

GraphPad Prism 9.3.0, Perl software (version 5.3), and R software (version 4.0.3) were utilized. This work used the LASSO method, PCA, ROC analysis, Kaplan-Meier method, univariate and multivariate Cox proportional hazards models, and WGCNA modular analysis. Furthermore, the ΔΔCT method was utilized to quantify the rt-qPCR results, and a Student’s *t*-test was employed for analysis.

### Data availability

The datasets analysed during the current study are available in the TCGA website (https://portal.gdc.cancer.gov/) repository.

## RESULTS

### Functional modules identified by WGCNA analysis

**Figure 1:**
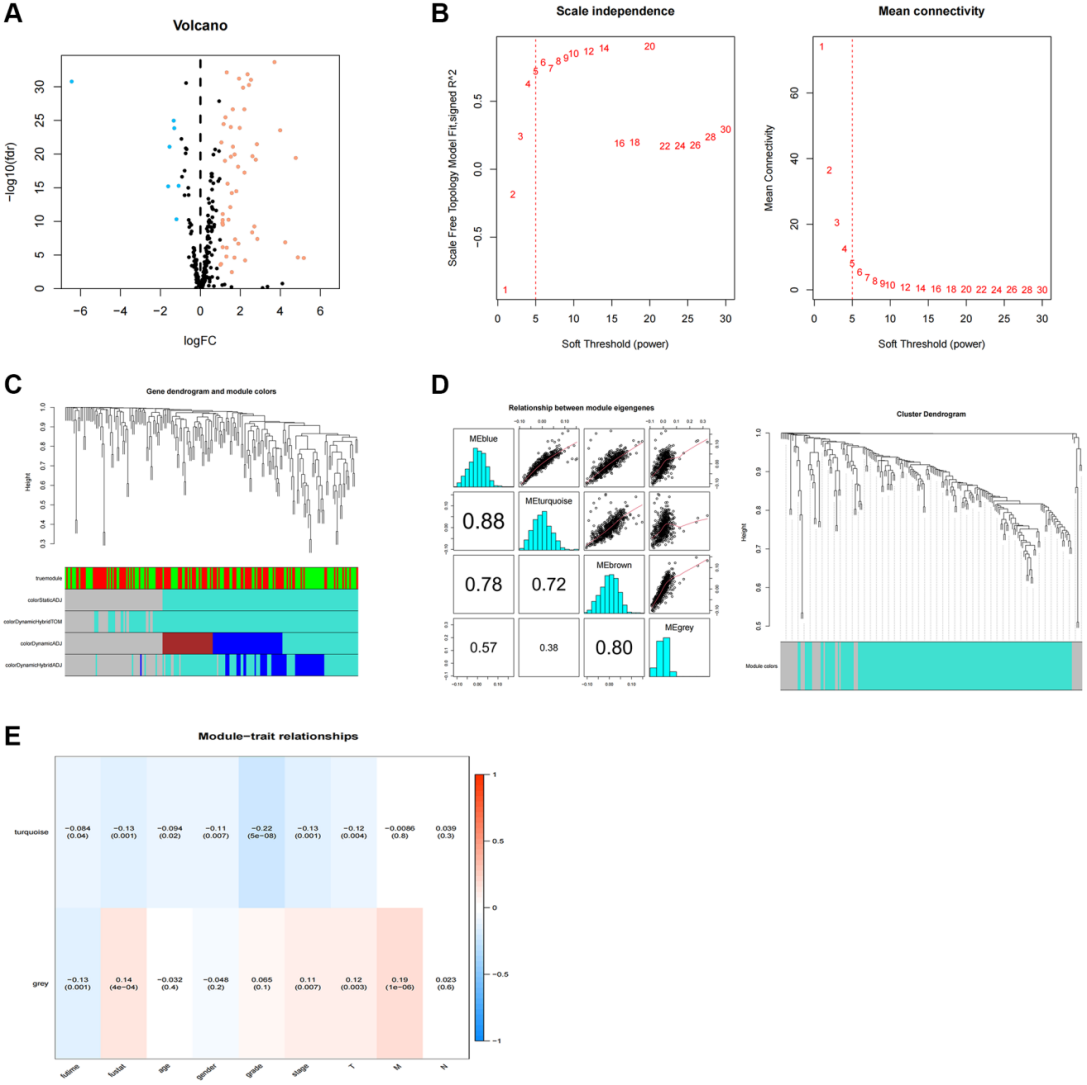
**WGCNA analysis of differentially expressed telomerase-associated genes.** (**A**) 256 TR-related genes in ccRCC. Yellow dots indicate up-regulated genes and blue dots indicate down-regulated genes. (**B**) Cluster analysis of samples. (**C**) Gene dendrogram and module colors. (**D**) Cluster-based gene dendrogram. (**E**) Correlations of modules with clinical phenotypes.

**Figure 2:**
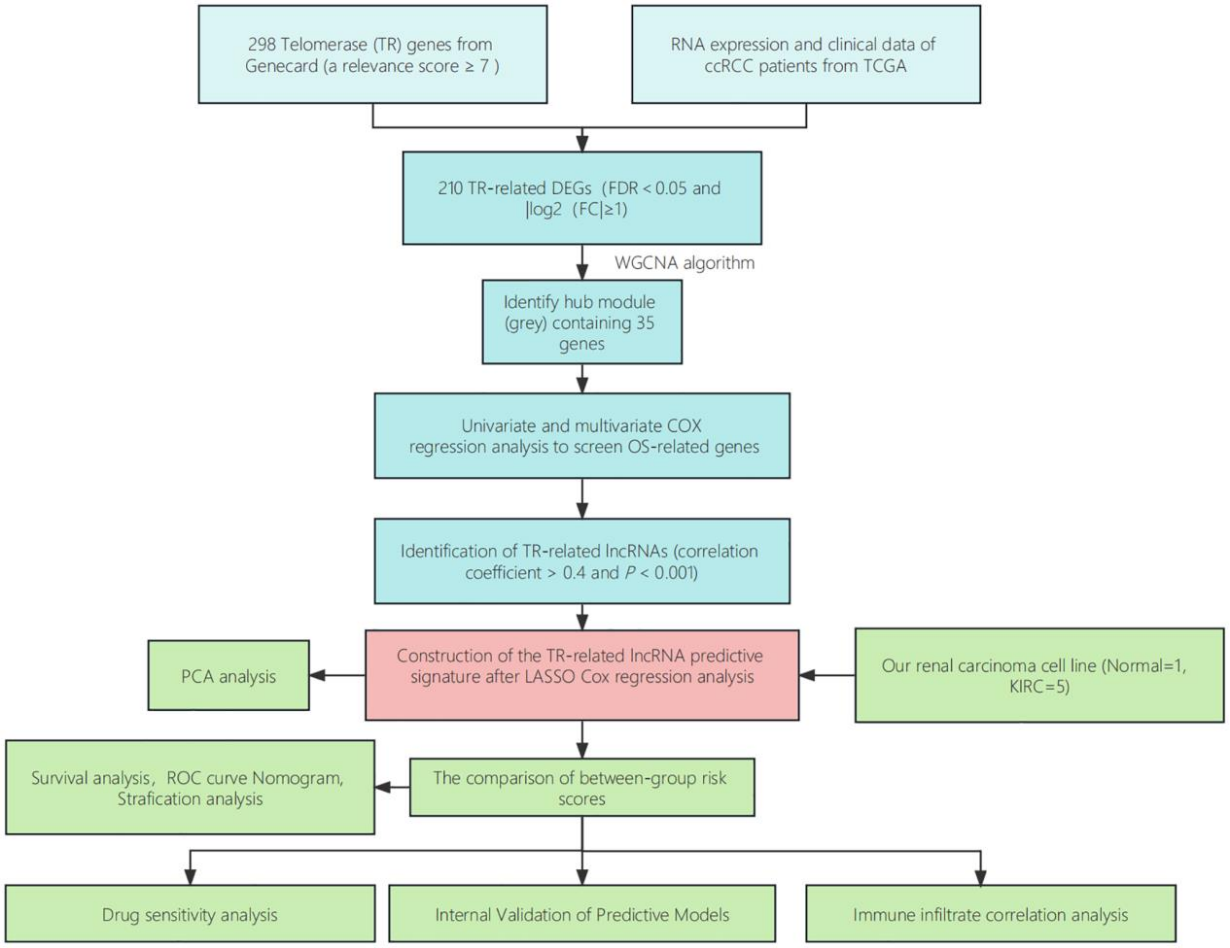
**Step diagram of the study.** Abbreviations: ccRCC: renal clear cell carcinoma; TCGA: Cancer Genome Atlas; DEGs: differentially expressed genes; lncRNAs: long non-coding RNAs; ROC: receiver operating characteristic; PCA: principal component analysis.

We screened 210 TR-associated DGEs (Figure 1A), adhering to the method illustrated in Figure 2. The selected DEGs were then applied to WGCNA. Using the genes’ expression data, the similarity matrix is first created by calculating the Pearson correlation coefficient between the two genes. Next, the similarity matrix is converted into an adjacency matrix, progressively transformed into a topology matrix that describes the degree of association to identify the differences between the genes using TOM. A power of β = 5 was used as the soft threshold parameter to meet the scale-free topology (R^2^ > 0.9) (Figure 1B). A hierarchical clustering tree was built using 1-TOM as the distance for gene clustering and a cutoff value of 0.25 (Figure 1C). Each module’s characteristic genes were determined, and adjacent modules were combined to create additional modules (Figure 1D). Ultimately, two gene modules were found (Figure 1E). The gray module showed a strong positive correlation with the scores on the clinicopathological index among them. As a result, 35 essential genes from the gray module were chosen for additional examination (Supplementary Table 2).

### Construction of prognostic risk models

**Figure 3:**
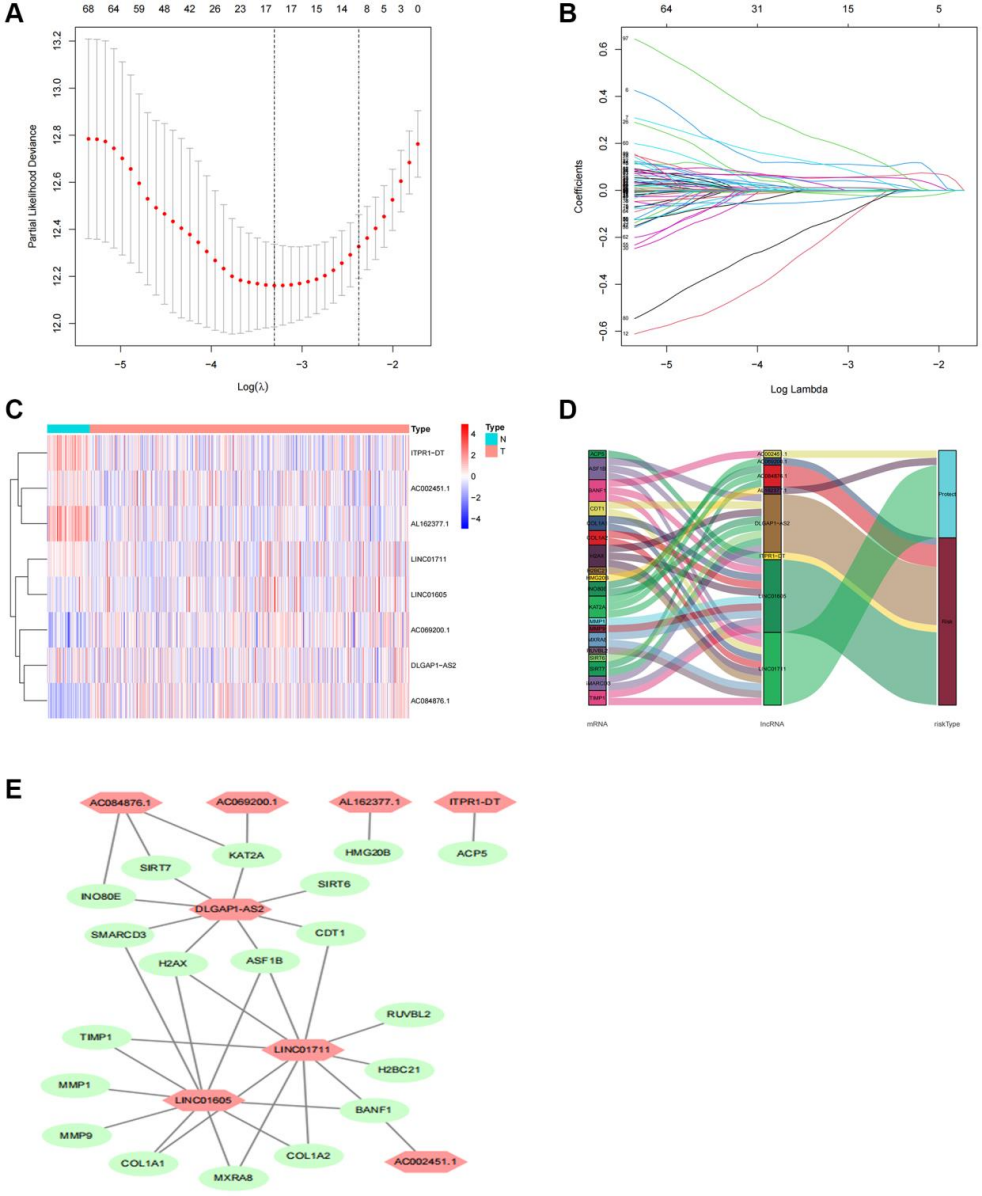
**Screening, expression levels, and lncRNA-mRNA networks of eight TR-related lncRNAs in predicted signals.** (**A**) Ten-fold cross-validation error rate plots. (**B**) LASSO coefficient profiles of TR-related lncRNAs. (**C**) Expression levels of eight TR-related lncRNAs in ccRCC and normal tissues. (**D**) Co-expression networks of prognostic TR-related lncRNAs. (**E**) Multinomial plots of prognostic TR-related lncRNAs. Abbreviations: lncRNAs: long-chain non-coding; ccRCC: renal clear cell carcinoma.

A total of 1892 lncRNAs linked to TRs with differential expression were screened (Supplementary Table 3). Further univariate Cox regression analysis yielded 99 lncRNAs associated with ccRCC patient prognosis (Supplementary Table 4). To predict the prognosis of patients with ccRCC, we employed LASSO Cox regression to build prognostic models utilizing prognosis-related lncRNAs and 1000 10-fold cross-validation (Figure 3A, 3B). The findings demonstrated that predictive characteristics were constructed using eight lncRNAs linked with TRs (AC069200.1, AC002451.1, LINC01711, ITPR1-DT, DLGAP1-AS2, AL162377.1, LINC01605, AC084876.1). Figure 3C displays the expression levels of eight lncRNAs in the expected signature. To see lncRNAs, we also used the R software tools gg alluvial and Cytoscape. The co-expression network displayed results for 33 pairs of lncRNA-mRNAs (Figure 3D). Protective factors were AC002451.1, LINC01711, and AL162377.1, while risk factors included AC069200.1, ITPR1-DT, DLGAP1-AS2, LINC01605, and AC084876.1 (Figure 3E). This is how the risk score was determined: Risk score is equivalent to the following: (0.30 × AC069200.1 expression) + (−1.39 × AC002451.1 expression) + (−0.45 × LINC01711 expression) + (0.66 × ITPR1-DT expression) + (0.48 × DLGAP1-AS2 expression) + (−1.05 × AL162377.1 expression) + (1.05 × LINC01605 expression) + (0.73 × AC084876.1 expression).

### Association between prognosis and prognostic characteristics in ccRCC patients

**Figure 4:**
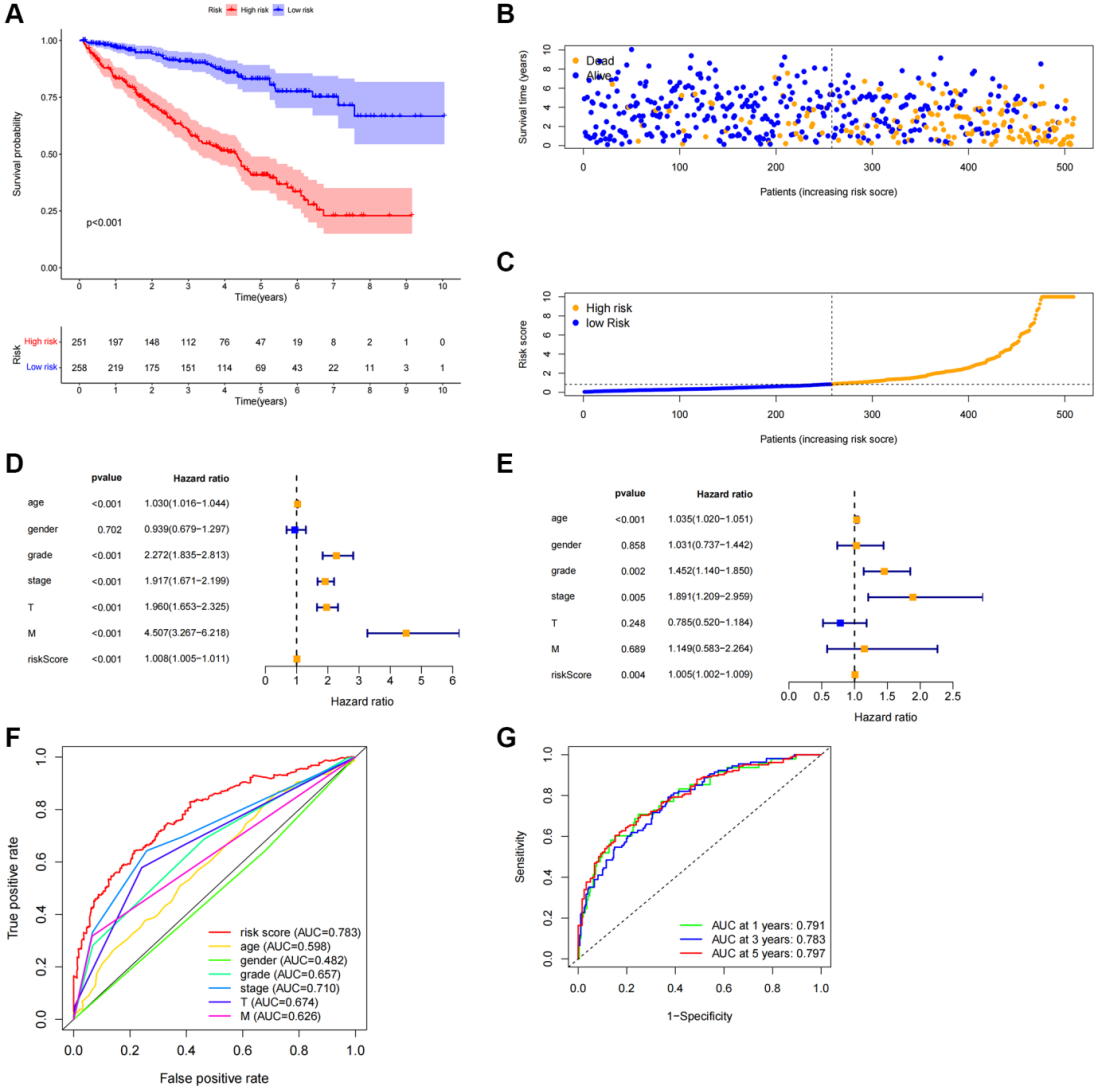
**Correlation between predictive characteristics and prognosis of ccRCC patients.** (**A**) Kaplan-Meier analysis of OS rate in patients with high and low risk. (**B**) Risk score distribution in patients with ccRCC. (**C**) The total number of patients with varying risk scores who died and survived. Red denotes the number of deaths, and blue is the number of survivors. (**D**) The univariate Cox regression analysis’s forest plot. (**E**) Multivariate Cox regression analysis’s forest plot. (**F**) Risk score and clinicopathological variable ROC curve. (**G**) The ROC curve and AUCs are used to estimate the survival rates of features after one, three, and five years. Abbreviations: ccRCC: renal clear cell carcinoma; OS: survival rate; ROC: receiver operating characteristic; AUC: area under the curve; T: tumor; M: metastasis.

**Figure 5:**
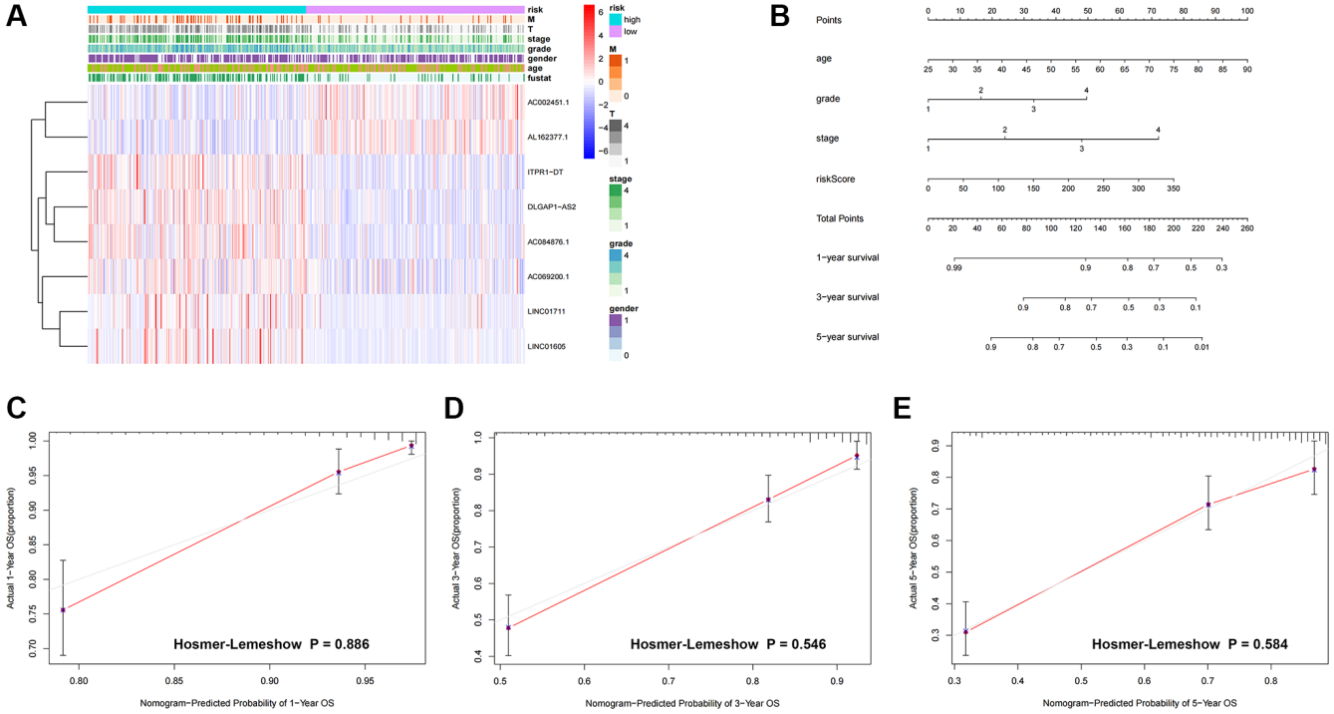
**Heatmap and nomogram construction and validation of the distribution of model signatures and clinicopathological variables.** (**A**) Heatmap showing the distribution of clinicopathological factors and eight lncRNAs associated with prognostication. (**B**) For ccRCC patients, survival at 1, 3 and 5 years was predicted by nomogram survival in conjunction with risk scores and clinicopathological factors. (**C**–**E**) Calibration curves and Hosmer-Lemeshow test for the validation of the predicted probability of 1, 3, 5-year survival. Abbreviations: lncRNAs: long-chain non-coding RNAs; T: tumor size; M: metastasis; OS: overall survival; ccRCC: renal clear cell carcinoma.

Each patient’s risk score was determined using the algorithm, and based on the median value, the patients were categorized as either high-risk or low-risk. The two groups’ overall survival times were compared using Kaplan-Meier analysis, and the findings indicated that the low-risk group’s OS was much shorter than the high-risk group’s (Figure 4A, *P* < 0.001). Figure 4B displayed the difference in the risk score, and Figure 4C showed that as the risk score increased, more deaths occurred. Univariate Cox regression analysis revealed that age, grade, stage, M stage, and risk score were significantly correlated with the OS of ccRCC patients (Figure 4D). Multivariate Cox regression analysis revealed that age, grade, stage, and risk score were independent predictors of OS of ccRCC patients (Figure 4E), indicating that the risk characteristics were independent risk factors for the prognosis of ccRCC patients. Compared to other clinicopathological characteristics, the risk score’s area under the curve (AUC = 0.783) was more predictive of a favorable outcome (Figure 4F). The 1-year, 3-year, and 5-year survival AUCs had predictive solid ability. According to Figure 4G, the areas were 0.791, 0.783, and 0.797, respectively. We evaluated the differences in clinicopathological variables between the high-risk and low-risk groups to rule out these variables’ influence, and we found no significant differences (Figure 5A).

We created nomogram prediction maps combining clinicopathological characteristics and risk scores to further forecast the prognosis of patients with colorectal cancer (ccRCC). These maps were used to predict the patients’ 1-year, 3-year, and 5-year prognoses (Figure 5B). For further evaluation, the accuracy of prognostic variables was assessed by calibration analysis and Hosmer-Lemeshow (HL) test. Consistent HL test statistics showed predictive probabilities for survival at 1 year (Figure 5C, *P* = 0.886), 3 years (Figure 5D, *P* = 0.546), and 5 years (Figure 5E, *P* = 0.580), signifying good calibration, indicating good model fit.

### Relationship between predictive characteristics and prognosis of different clinicopathologic parameters in ccRCC patients

**Figure 6:**
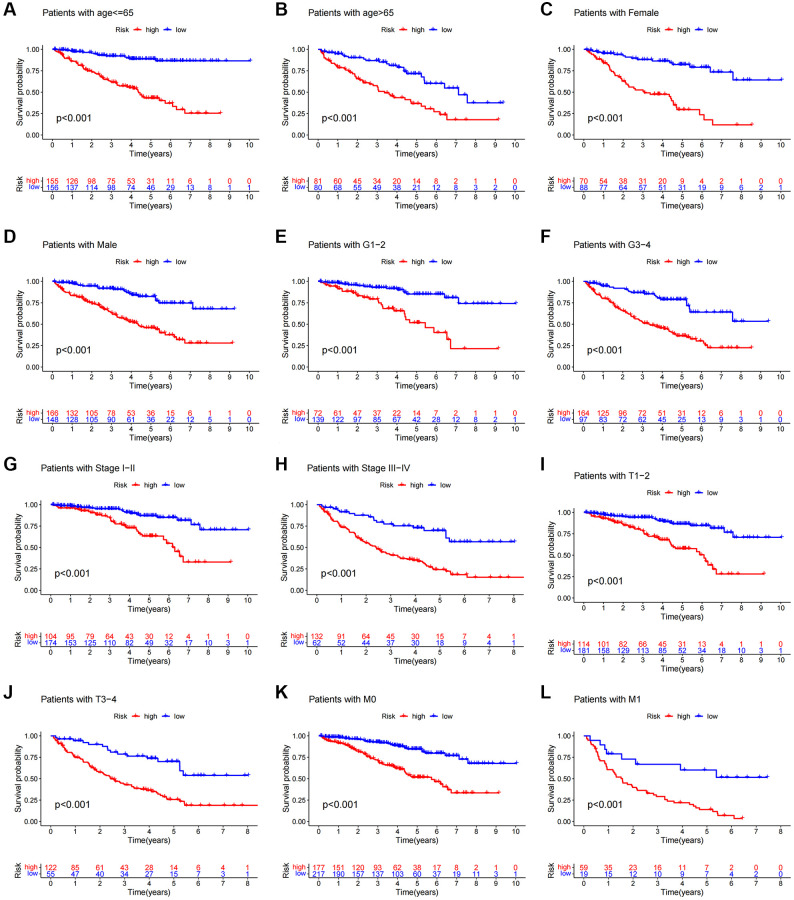
**Kaplan-Meier survival curves of patients divided into high- and low-risk groups according to the ranking of different clinicopathological variables.** (**A**, **B**) Age. (**C**, **D**) Gender. (**E**, **F**) Grade. (**G**, **H**) Stage. (**I**, **J**) T Stage. (**K**, **L**) M Stage. T, tumor size. M, distant metastasis.

We carried out a subgroup analysis of survival in patients with varying ages, pathological kinds, grades, and M and N stages to examine the association between predictive indicators and prognosis in ccRCC patients under various clinicopathological variable classifications. According to the results, OS in the high-risk group was considerably worse than in the low-risk group (Figure 6), suggesting that the predictive features might be used to forecast the prognosis of patients with colorectal cancer under various clinicopathological conditions.

### Performing internal validation of predictive features

**Figure 7:**
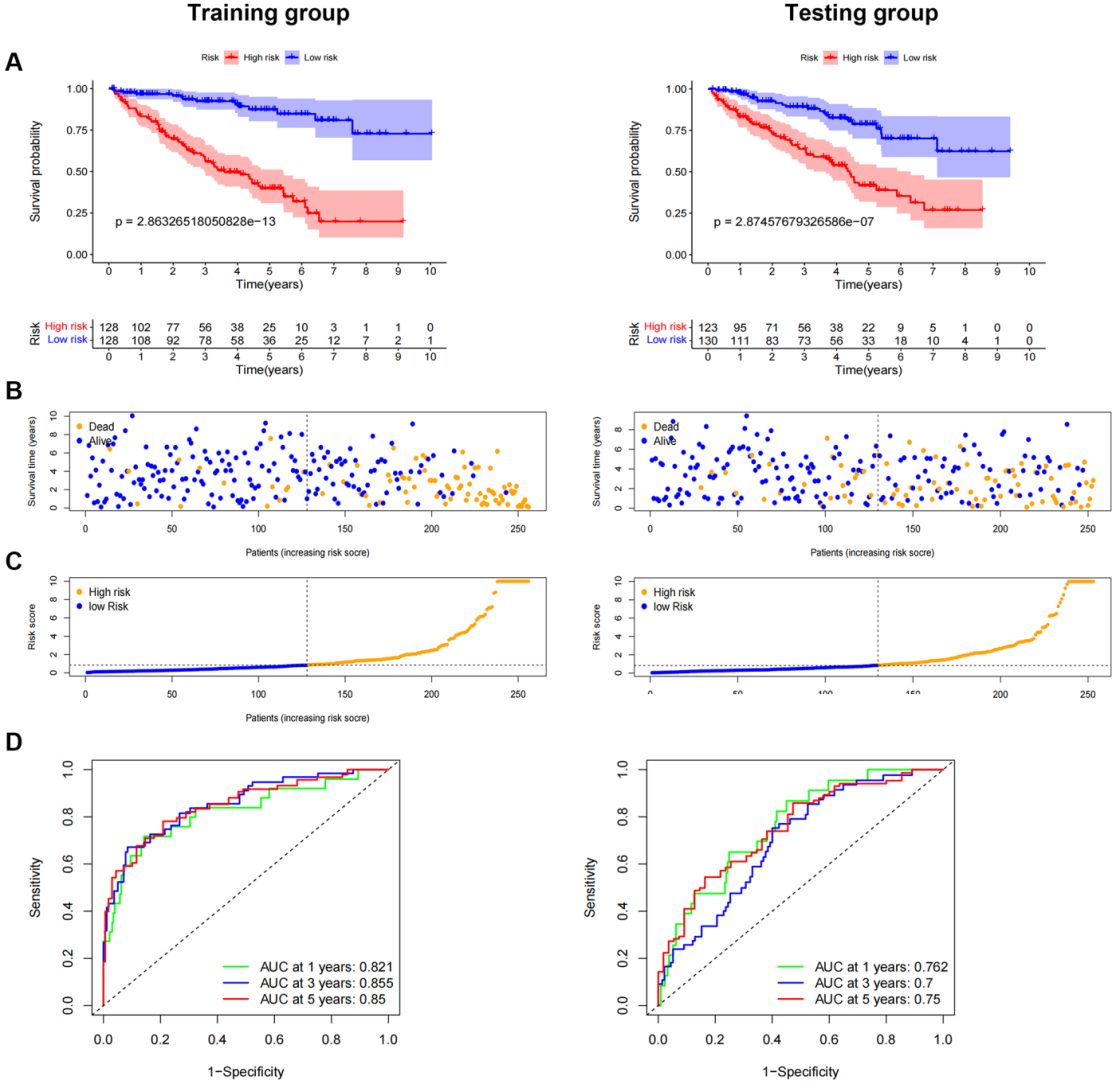
**Internal validation of OS prediction signatures based on internal datasets.** Kaplan-Meier survival curves for the test and training groups. (**A**, **B**) Training and test groups’ survival duration and status. (**C**) Training and test groups’ distribution of total survival risk scores. (**D**) ROC curves and AUCs for patients in the training and test groups’ 1-, 3-, and 5-year survival rates. Abbreviations: ROC: receiver operating characteristic; AUC: area under the curve; OS: overall survival; TCGA: Cancer Genome Atlas.

We randomly split ccRCC patients into two groups to test the applicability of the prognostic signature based on the whole TCGA dataset. All high-risk patient groups in the training and validation groups had considerably lower overall survival rates, consistent with the findings across the board (Figure 7A–7C). The patients’ clinical manifestations were visible by examining the ROC curves of the two groups. The training group’s 1-, 3-, and 5-year survival rates had AUCs of 0.821, 0.855, and 0.850, respectively (Figure 7D). The validation group’s 1-, 3-, and 5-year survival rates had AUCs of 0.762, 0.700, and 0.750, respectively (Figure 7D).

### Comparison of prognostic features based on gene expression in renal clear cell carcinoma

Advances in big data and next-generation sequencing technology have led to the emergence of major machine learning-based prognostic and predictive gene expression signatures in recent years [24]. To compare performance with other signatures, we thoroughly searched ccRCC-released signatures. Six signatures have been registered [25–30] (Supplementary Table 5). These characteristics include pyroptosis, iron death, RNA-binding protein, necroptosis, WNT, and drug-sensitive biological processes. Using the ccRCC’s time-dependent ROC, we looked at every model to forecast its predictive impacts. Other models are not as good as our TRL model.

### Immune cell infiltration and functional analysis

**Figure 8:**
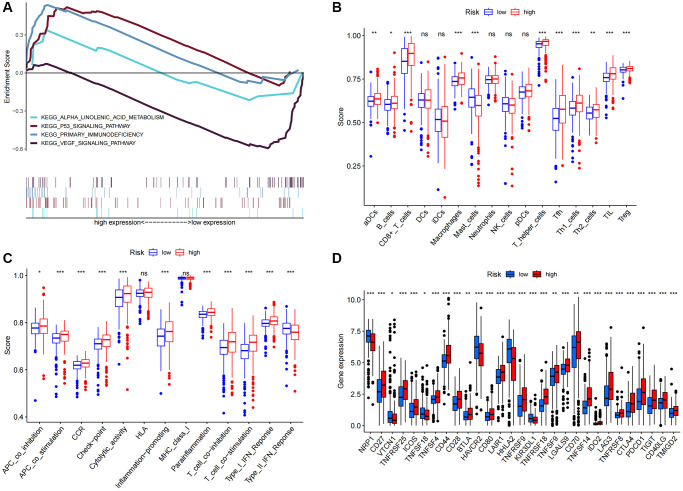
**KEGG and immune infiltration analysis.** (**A**) KEGG enrichment analysis. (**B**) 16 immune cell scores. (**C**) 13 immune-related function scores. (**D**) Expression of immune checkpoints in high- and low-risk populations. Abbreviations: KEGG: Kyoto Encyclopedia of Genes and Genomes ssGSEA; single-sample gene set enrichment analysis; aDCs: activated dendritic cells; iDCs: immature dendritic cells; NK: natural killer cells; pDCs: plasmacytoid dendritic cells; Tfh: T follicular helper cells; Th1: T helper type 1; Th2: T helper type 2; TIL: tumor-infiltrating lymphocytes; Treg: T regulatory cells; APC: antigen-presenting cells; CCR: chemokine receptor; HLA: human leukocyte antigen; MHC: major histocompatibility complex; IFN: interferon. ^*^p < 0.05; ^*^p < 0.01; ^**^p < 0.001; Abbreviation: ns: not significant.

Upon conducting additional KEGG analysis, we discovered that genes associated with the prognostic signature risk model were abundant in signaling pathways related to immune response, p53 signaling pathways, VEGF, and α-linoleic acid metabolism (Figure 8A). We used ssGSEA to quantify enrichment scores for various immune cell subsets, related functions, or pathways to explore better the relationship between risk scores and immune cells and function (Figure 8). The findings demonstrated significant differences between patients in the high and low-risk groups in terms of activated dendritic cells (aDCs), CD8 + T cells, macrophages, mast cells, T helper cells, T follicular helper (Tfh), T helper type 1 (Th1), T helper type 2 (Th2), tumor-infiltrating lymphocytes (TIL), and regulatory T cells (Treg) (Figure 8C). The high-risk group had higher levels of antigen-presenting cell (APC) co-inhibition, APC co-stimulation, chemokine receptor (CCR), checkpoint, cytolytic activity, pro- and para-inflammatory, T cell co-inhibition, T cell co-stimulation, and immune function scores of type I IFN response, indicating active immune function. Subsequent investigation demonstrated that the immune checkpoint expressions of the two groups also differed (Figure 8D).

**Figure 9:**
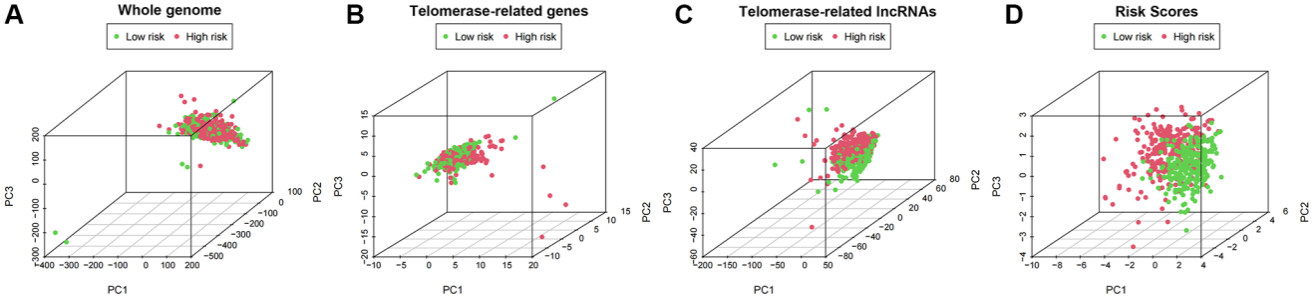
PCA profiles showed patient distribution based on (**A**) Genome-wide; (**B**) Telomerase-related genes; (**C**) Telomerase-related lncRNAs; and (**D**) Risk score. In the high- and low-risk groups, red and green dots were more strongly separated.

We employed principal component analysis (PCA) to display the distribution of patients based on genome-wide, TR-related gene sets, TR-related lncRNAs, and predictive characteristics to visualize the spatial distribution of high-risk and low-risk groups. The findings demonstrated that when predictive indicators were used to separate patients, patients in the high-risk group were dispersed over several quadrants (Figure 9A–9D).

### Predictive characteristics associated with ccRCC therapy

**Figure 10:**
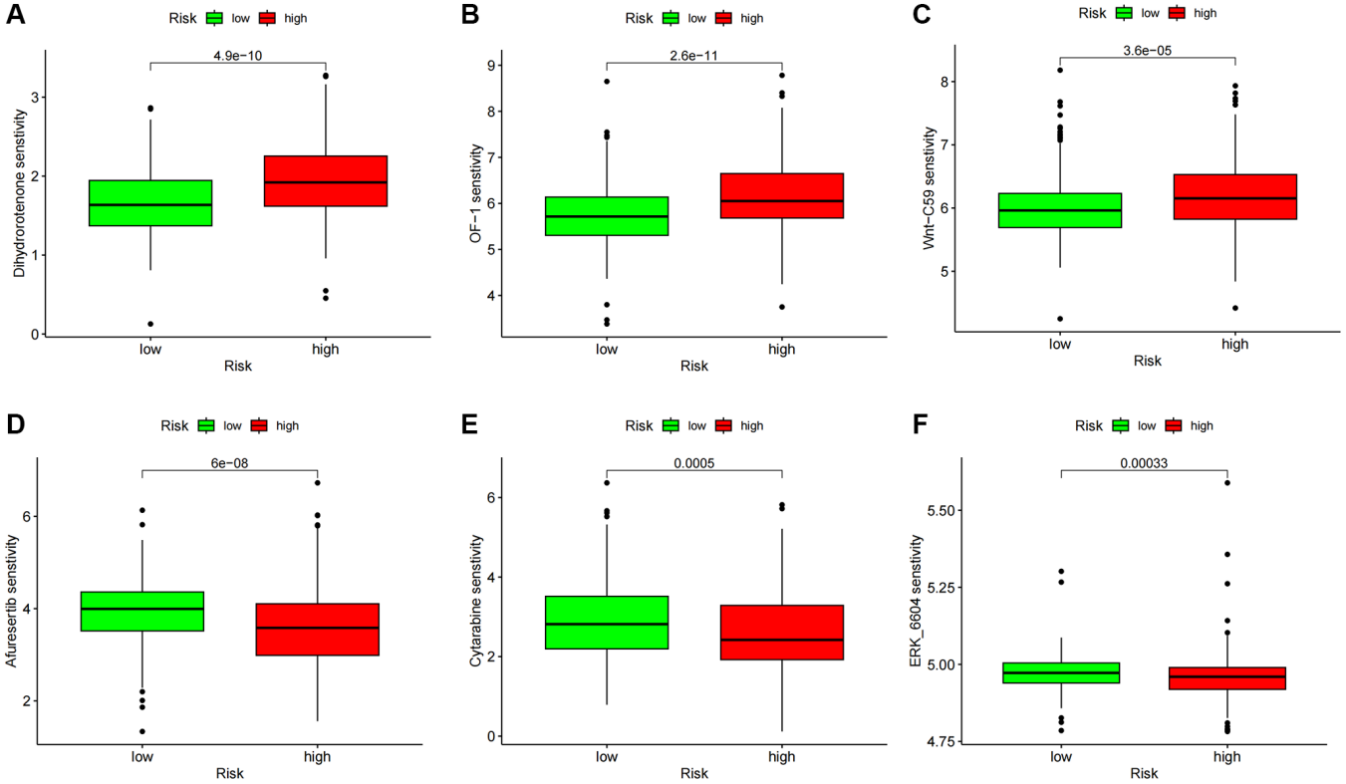
**Drug sensitivity analysis.** (**A**–**C**) Predicted sensitivity scores Dihydrorotenone, OF-1, and Wnt-C59 were candidates for chemotherapy in patients with high-risk scores. (**D**–**F**) Afutib, Cytarabine, and ERK6604 were candidates for chemotherapy in patients with high-risk scores. ^*^p < 0.05; ^*^p < 0.01; ^*^p < 0.0001.

The OncoPredict program was utilized to forecast high- and low-score drug sensitivity scores. The results showed a positive correlation between sensitivity scores and IC_50_ values of chemotherapeutic drugs. In the low-score group, dihydro rotenone, OF-1, and Wnt-C59 exhibited increased sensitivity (Figure 10A–10C). The high-risk group showed increased sensitivity to afuresertib, cytarabine, and ERK6604 (Figure 10D–10F). Examining specific chemotherapy regimens that are appropriate for people in the high- and low-risk categories is beneficial.

### Expression of risk model lncRNAs

**Figure 11:**
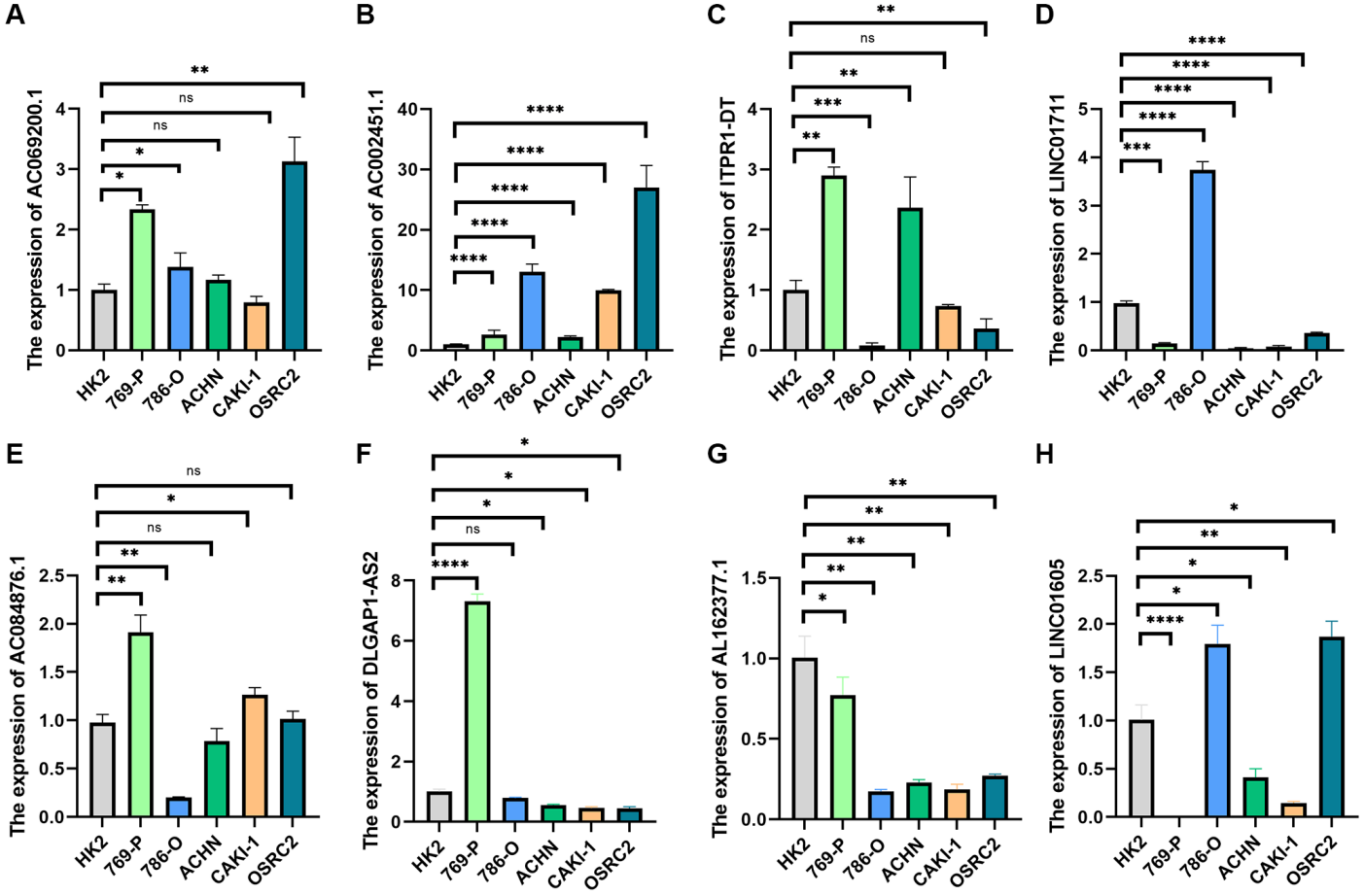
**qRT-PCR was used to detect lncRNA expression levels in cancer cell risk models (HK2, 769-P, 786-O, ACHN, CAKI-1, and OSRC2).** (**A**) AC069200.1; (**B**) AC002451.1; (**C**) ITPR1-DT; (**D**) LINC01711; (**E**) AC084876.1; (**F**) DLGAP1-AS2; (**G**) AL162377.1; (**H**) LINC01605. ^*^p < 0.05; ^*^p < 0.01; ^*^p < 0.001.

Further research was done on the expression of AC069200.1, AC002451.1, LINC01711, ITPR1-DT, DLGAP1-AS2, AL162377.1, LINC01605, and AC084876.1. Eight lncRNAs were tested for expression using human renal cell carcinoma cell lines (769-P, 786-O, ACHN, CAKI-1, and OSRC2) and tubular epithelial cells (HK2). All eight lncRNAs were expressed differently in ccRCC cell lines compared to tubular epithelial cell lines, according to a quantitative real-time PCR (qRT-PCR) investigation (Figure 11A–11H). These findings imply that eight lncRNAs might be significant in ccRCC. We further analyzed the correlation between these clinical variables and the risk scores of the eight lncRNAs based on the gene expression and corresponding clinical data obtained from the TCGA database. The findings indicated that the eight lncRNAs and risk scores were associated with age, gender, tumor stage, and grade (Supplement Figure 1).

### Assessment of biological function for AC002451.1

**Figure 12:**
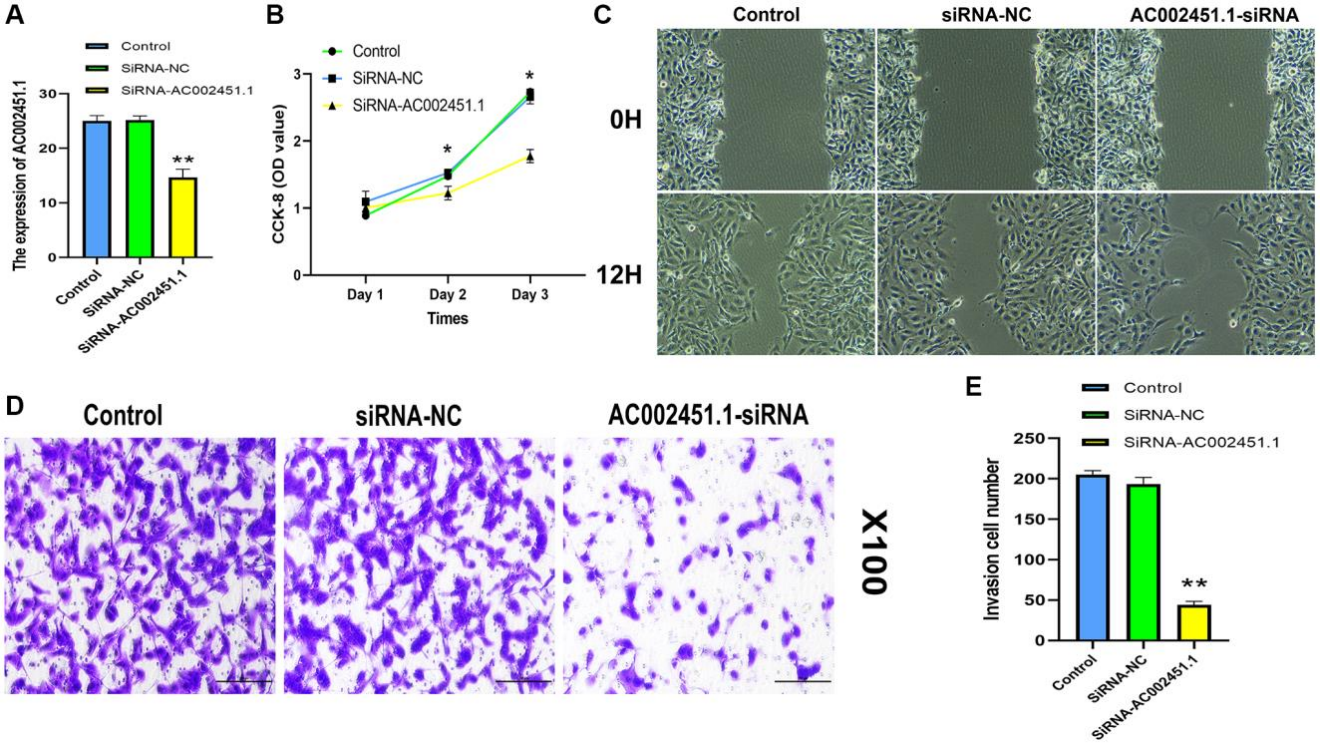
**Assessment of biological function for AC002451.1.** (**A**) RT-PCR validation following AC002451.1 knockdown in OSRC2 cells. (**B**) CCK-8 assay was performed to determine the proliferation ability of OSRC2 cells after AC002451.1 knockdown. (**C**) A wound healing assay was used to assess the migration ability of cells following AC002451.1 knockdown. (**D**, **E**) Transwell assay was used to assess the migration ability of cell lines following AC002451.1 knockdown. ^*^p < 0.05, ^*^p < 0.01, ^**^p < 0.001, ^*^p < 0.0001.

We observed that AC002451.1 showed a significant difference between renal cancer and adjacent non-cancerous tissues, and the expression level was the highest in the OSRC2 cell line, so we successfully interfered with AC002451.1 mRNA expression using siRNA technology in the OSRC2 cell line and confirmed it by RT-PCR (Figure 12A). CCK-8 assay results showed cell viability inhibited after silencing AC002451.1 expression (Figure 12B). To further understand the effect of AC002451.1, colony formation assays were also performed, and the results showed that the viability of renal cancer cells was significantly inhibited after AC002451.1 was knocked down by siRNA (Figure 12C). To further examine whether AC002451.1 affected renal clear cell carcinoma metastasis, we performed transwell assays to assess the migration and invasion of renal carcinoma cells. These results indicate that siRNA-mediated silencing of AC002451.1 suppressed invasion of renal clear cell carcinoma (Figure 12D, 12E).

## DISCUSSION

The urothelial system of the renal parenchyma is affected by kidney renal clear cell carcinoma (KIRC), an illness with a highly complicated etiology. The majority of treatment for KIRC is surgery because the condition is typically resistant to both chemotherapy and radiation. Thirty percent of tumor patients will eventually develop metastases, even with early surgical intervention [31]. As bioinformatics technology develops, more biomarkers will be found and could be helpful as targets for ccRCC diagnosis and treatment. Although specific biomarkers can increase the prediction findings’ accuracy [32, 33], single molecular markers cannot match our standards for outcome prediction due to the heterogeneity of colorectal cancer (ccRCC) disease. Multivariate model building for cancer prognostic prediction has emerged as a significant area of scientific interest.

It has been documented that cancer cells use telomerase or telomere-selective elongation (ALT) activation to preserve telomere length and accomplish immortality [34]. While most normal somatic tissues lack telomerase, most human malignancies express high quantities of this enzyme and have short telomeres [35, 36]. TERT plays a significant role in cancer formation, which is a limiting factor in the production of telomerase complexes in cancer cells [37]. The catalytic protein of telomerase RNP, hTERT, is a crucial step in generating telomerase activity [38]. Chromosome rearrangements at the TERT locus have been linked in several studies to the development of neuroblastoma [39–41]. Thyroid cancer, particularly papillary thyroid cancer patients with a poor prognosis, can also be caused by TERT promoter (TERTp) mutations in conjunction with BRAF mutations [42, 43]. Additionally, it has been discovered that the TERT hypermethylation tumor region (THOR), which is linked to elevated TERT expression and accelerates the development of pancreatic and stomach tumors, is present in these diseases. According to one study, the risk of RCC may be related to the number of polymorphisms in tandem repeats in human telomerase reverse transcriptase (hTERT) MNS16A, rs2736098 [44]. Telomerase’s function in different cancers has been the subject of countless investigations, although its significance in ccRCC is still unclear. Finding important telomerase-related molecular markers and investigating their function in the growth of ccRCCs may shed light on the biological characteristics of these cancers and suggest fresh, more potent treatment approaches.

From the TCGA database, we extracted clinical and RNA-seq data from 72 standard samples and 539 ccRCC samples. WGCNA modular analysis was used to identify module genes strongly correlated with patient clinicopathology. Cox and Lasso regression analyses were then used to select eight lncRNAs to establish a risk score model, which classified ccRCC patients into low- and high-risk groups based on the model’s risk score. PCA demonstrated that risk ratings may be used to separate patients into two groups. According to Kaplan-Meier survival analysis, patients in the high-risk group had a lower overall survival (OS) than those in the low-risk group. Moreover, scatter plots of survival status indicated a negative correlation between risk scores and survival. Univariate and multivariate Cox analyses demonstrated independent predictive risk factor status for the risk score. According to survival data, patients in the high-risk category have a terrible prognosis when compared to low-risk patients in various clinical groupings. These findings verified the predictive utility of this model throughout training, testing, and the whole TCGA cohort. Furthermore, we discovered that the risk score was connected to clinical traits, indicating that the model might be applied as a predictive and diagnostic tool. Our next step was to create a nomogram with risk scores to forecast the 1-, 3-better, and 5-year survival of patients with colorectal cancer. The nomogram’s ROC curve shows that its accuracy is relatively high. Differentially expressed genes in risk categories were shown to be considerably enriched in signaling pathways like P53 and immunology, according to functional enrichment analysis. Furthermore, there were notable differences in the activation of many immunological pathways between the high-risk and low-risk groups. It has been steadily demonstrated that lncRNA plays a role in tumor growth and functions as an immunotherapy predictor [45–47]. Thus, more research is required on the connection between immunotherapy and TR-associated lncRNAs.

Tumor starts growth, and metastases are intimately linked to TME [48]. Long noncoding RNAs are a group of endogenous nonprotein-coding RNAs that regulate many cellular processes, including proliferation, differentiation, migration, and invasion. They also participate in innate and adaptive immunity by mediating TME [49]. According to specific research, lncRNAs may have a role in controlling TME in ccRCC [50]. For instance, immune checkpoint expression and immune cell infiltration in ccRCC are believed to be related to lncRNA MIR155HG [51]. Furthermore, a growing number of irlncRNAs have been employed for significant and encouraging prognostic prediction in patients with colorectal cancer (ccRCC), including the possible therapeutic benefit of immunotherapy [52, 53]. Pyroptosis is a special type of programmed cell death involved in tumorigenesis, progression, immune infiltration, and anti-tumor responses [54]. A recent study showed that ccRCC with different pyroptotic states showed significant heterogeneity at multiple levels, including functional status and tumour microenvironment [55]. However, this failed to reveal the role of pyroptosis-associated lncRNAs in regulating ccRCC immune regulation. Our GSEA results strongly correlated with tumor and immune-related pathways and high-risk patients. The high-risk group scored higher for CD8 + T cells, macrophages, Tfh cells, TILs, and Tregs, according to subsequent ssGSEA data. Research has demonstrated that a significant infiltration of CD8 + T cells is linked to a poor prognosis for BC patients [56, 57]. In advanced thyroid carcinoma, a high infiltration of tumor-associated macrophages is linked to a poor prognosis [58]. Patients with HCC who have a high infiltration of Tregs have a bad prognosis [59]. The high-risk group exhibited higher type I IFN response scores, lower antitumor immune capacity, and increased tumor immune cell infiltration. Consequently, a poor prognosis in high-risk groups may be caused by reduced antitumor immunity. According to our research, Sufuresertib, Cytarabine, and ERK6604 are standard chemotherapy drugs that may be sensitive to high-risk patients. This demonstrates that immunotherapy plus chemotherapy can be beneficial for high-risk groups, offering a foundation for accurate, individualized treatment of patients with colorectal cancer.

Our research creates a risk model that can be used in clinical settings and highlights the significant role that telomerase-associated lncRNAs play in ccRCC. A series of correlation analyses were used to determine the clinical utility of the model, which classified ccRCC patients into several risk groups. Our study does, however, have a few drawbacks. Firstly, we employed only TCGA data for model creation and validation because no large cohorts containing gene expression and clinical information were found. As a result, assessing the risk model’s applicability in additional datasets was required. Furthermore, our study is based on a genomics-level single survey. Meng et al. [60] recently delineated the multi-body subtype (MoS) classification and proposed a multi-omics-based ccRCC classification system scheme, promoting further biological understanding of ccRCC. Finally, to confirm the mechanism of action of these model genes in ccRCC, additional *in vitro* investigations are necessary because of the limitations of our current *in vitro* studies.

## CONCLUSION

To sum up, the features of the TRL model can independently predict the prognosis of patients with colorectal cancer. They can also offer a potential method for choosing chemotherapeutic and anti-tumor immunotherapy medications.

## Supplementary Materials

Supplementary Figure 1

Supplementary Tables 1-2 and 4-5

Supplementary Table 3
