# Supplementary material for: LASSO regression and WGCNA-based telomerase-associated lncRNA signaling predicts clear cell renal cell carcinoma prognosis and immunotherapy response
Source: Aging (Albany NY). 2024 May 30;16(11):9386–409. doi: 10.18632/aging.205871 (PMC11210217; doi:10.18632/aging.205871)
Supplement: Supplementary Figure 1 [file aging-16-205871-s001.pdf]

## SUPPLEMENTARY FIGURE

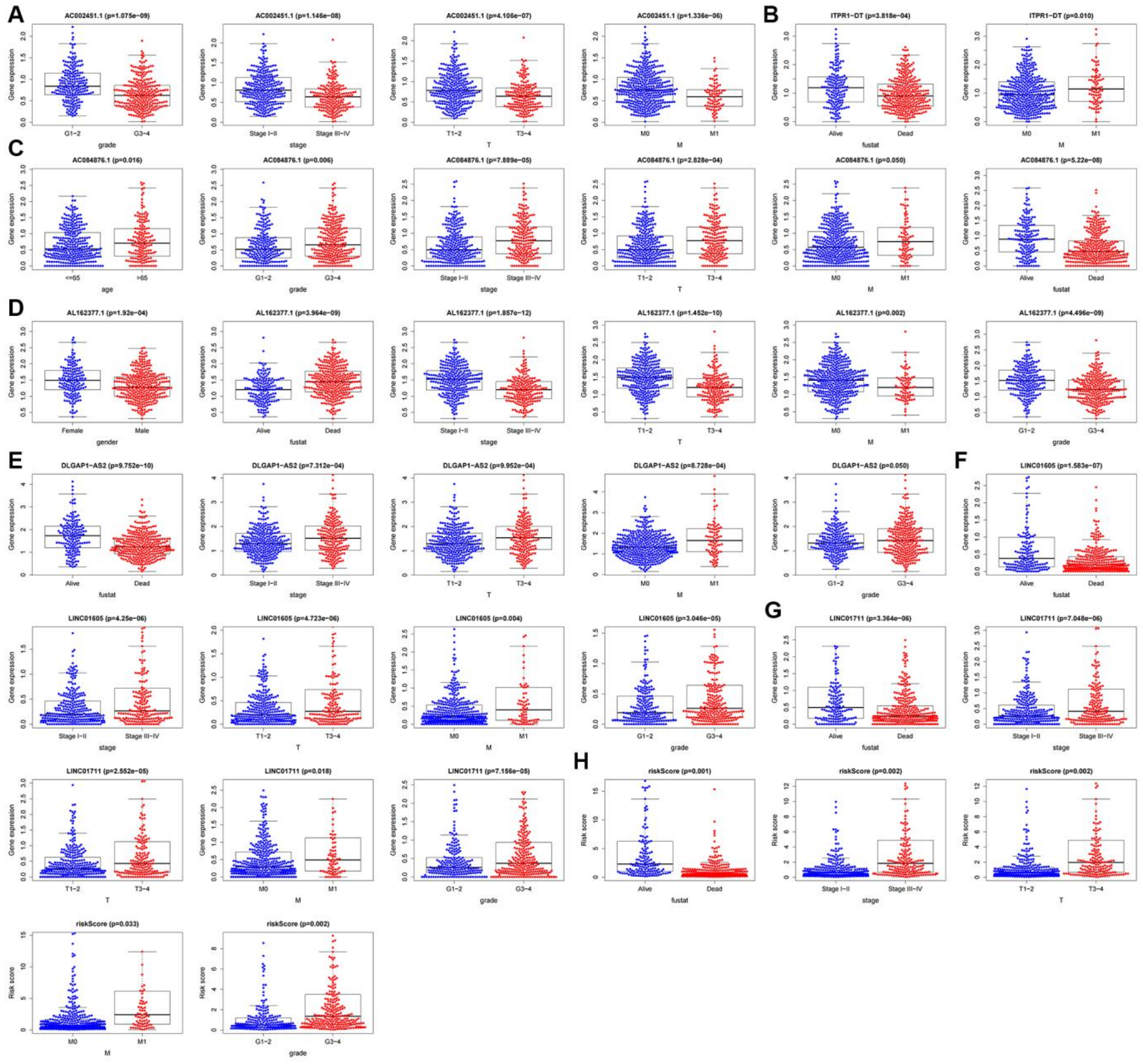


**Supplementary Figure 1. Correlation analysis between the model and clinical characteristics.** (**A**) Correlation between AC069200.1 expression level and grade, stage, and T stage. (**B**) Relationship between ITPR1-DT expression level and Survival status, and, M stage. (**C**) Relationship between AC084876.1 expression level and age, gender, stage, Survival status, and grade. (**D**) Relationship between AL162377.1 expression level and gender, Survival status, tumor grade, T stage, M stage, and stage. (**E**) Correlation between DLGAP1-AS2 expression level and Survival status, T stage, M stage, grade and stage. (**F**) Relationship between LINC00460 expression level and Survival status, T stage, M stage, grade and stage. (**G**) Relationship between LINC01605 expression level and Survival status, T stage, M stage, grade and stage. (**H**) Relationship between riskScore expression level and Survival status, T stage, M stage, grade and stage.
