# Supplementary material for: LASSO regression and WGCNA-based telomerase-associated lncRNA signaling predicts clear cell renal cell carcinoma prognosis and immunotherapy response
Source: Aging (Albany NY). 2024 May 30;16(11):9386–409. doi: 10.18632/aging.205871 (PMC11210217; doi:10.18632/aging.205871)
Supplement: Supplementary Tables 1-2 and 4-5 [file aging-16-205871-s002.pdf]

SUPPLEMENTARY TABLES

Supplementary Table 1. Sequences of upstream and downstream primers of prognostic model genes.

Primer Name	Primer sequences (5′–3′)	Fragment length (bp)	Annealing temperature (℃)	GC%	Number of bases	nmol/ OD
AC069200.1-S	CAAGGGCAAAGAAGGCACA	83	59.3	52.6	19	4.20
AC069200.1-A	AAGTCAGCCTCATTCCATACCC		59.9	50	22	4.43
AC002451.1-S	GTGGGAAGAACAGGGAAGAGG	208	60.1	57.1	21	3.73
AC002451.1-A	CAATGAGCAGGAATAGTAAGGGAG		59.6	45.8	24	3.33
ITPR1-DT-S	CAGTGTTTGCGGTCAAGGATT	210	60.1	47.6	21	4.40
ITPR1-DT-A	TATGGCAGATGCGGCAGTTT		61.5	50	20	4.58
DLGAP1-AS2-S	GACACAGACAAGACCCTTTCAATAA	181	59.24	40	25	3.43
DLGAP1-AS2-A	GACAGCATCGGGTCAAAGGA		60.04	55	20	4.22
AL162377.1-S	GTCTCCAGCAAGCGCAGTCA	113	62	60	20	4.69
AL162377.1-A	GTCGCAGAACCAGTGGCAAA		61.7	55	20	4.30
AC084876.1-S	ATGGTGCTGGATGGATTAGAGTT	294	59.6	43.5	23	3.84
AC084876.1-A	CATTGAATGGGTTTGCTACTGA		58	40.9	22	4.17
LINC01711-S	AGTTCGGGCAGCCATAGAGG	107	61.69	60	20	4.42
LINC01711-A	AGTGTTTCCAGCCATCAGGTT		59.85	47.62	21	4.58
LINC01605-S	CCGTTACAAACAGCCGACCTT	268	61.14	52.38	21	4.52
LINC01605-A	TGCAGGCTCCATTTCCAGAC		60.32	55	20	4.91
H-GAPDH-S	GGAAGCTTGTCATCAATGGAAATC	168	62.4	57.1	24	3.63
H-GAPDH-A	TGATGACCCTTTTGGCTCCC		62	55	20	5.25

Supplementary Table 2. Grey module gene.

Gene	Module
H19	grey
MBD3	grey
COL1A2	grey
FOS	grey
BANF1	grey
SPP1	grey
H3C6	grey
SIRT6	grey
BCL7C	grey
COL1A1	grey
H1-2	grey
ASF1B	grey
HMG20B	grey
TNFRSF11B	grey
MXRA8	grey
RUVBL2	grey
MMP9	grey

H2AX	grey
CDT1	grey
VDR	grey
MMP1	grey
ZNHIT1	grey
KAT2A	grey
JUN	grey
TIMP1	grey
SIRT7	grey
INO80C	grey
ACP5	grey
INO80E	grey
H4C8	grey
H4C9	grey
SMARCD3	grey
H2BC21	grey
H3C10	grey
H2BC12	grey

Supplementary Table 4. 99 lncRNAs associated with the prognosis of ccRCC patients by univariate Cox regression analysis.

Gene	KM	B	SE	HR	HR.95L	HR.95H	*p*-value
AL162458.1	1.56E-05	0.210649329	0.042290881	1.234479383	1.136281086	1.341164052	6.33E-07
AC124319.1	8.17E-06	0.178505579	0.039318739	1.195429552	1.106766019	1.291195963	5.63E-06
AC011462.4	7.40E-05	0.062837187	0.012284678	1.064853453	1.039520617	1.090803645	3.14E-07
AC004034.1	8.17E-05	0.29921675	0.06189628	1.348801944	1.194708558	1.52277028	1.34E-06
AC005785.1	7.93E-06	0.382610412	0.067190457	1.466106742	1.285206669	1.672469518	1.24E-08
AC016773.1	1.44E-06	0.589056386	0.071679545	1.802286949	1.566066495	2.074138138	2.07E-16
AC069200.1	1.12E-06	0.277641851	0.053350219	1.320013351	1.18895806	1.465514474	1.95E-07
AC005387.1	4.02E-07	0.213552338	0.040016348	1.238068295	1.144676126	1.339080171	9.47E-08
U52111.1	5.32E-06	0.314652283	0.062475652	1.369782931	1.211915608	1.548214467	4.74E-07
AL354760.1	3.58E-05	0.406129335	0.060619349	1.500996671	1.33284745	1.690359242	2.09E-11
TMEM92-AS1	2.29E-07	0.154742427	0.025593047	1.167357243	1.110245295	1.227407078	1.48E-09
AC002451.1	7.34E-05	−1.074856004	0.215923	0.341346904	0.223564055	0.521182661	6.43E-07
EMX2OS	3.10E-08	−0.028691561	0.004703093	0.971716133	0.962800115	0.980714719	1.06E-09
AP002807.1	1.12E-05	0.124064217	0.020185807	1.132088568	1.088173673	1.177775715	7.94E-10
AC009093.6	7.94E-05	0.273437337	0.061192657	1.314474987	1.165910041	1.481970677	7.88E-06
AC008537.4	6.35E-06	0.285509542	0.061348181	1.33043977	1.179710793	1.500427049	3.26E-06
AC006272.1	8.67E-06	0.375439593	0.090152948	1.45563116	1.219868496	1.736959418	3.12E-05
RNF139-AS1	3.49E-05	0.553654627	0.101568316	1.739599	1.425588035	2.122776431	5.01E-08
ZNF436-AS1	2.38E-05	0.138254947	0.031974331	1.148268261	1.078516419	1.222531224	1.53E-05
VPS9D1-AS1	3.03E-05	0.163556805	0.026836948	1.177692252	1.117347254	1.241296325	1.10E-09
AL158151.4	9.18E-07	0.221576207	0.035349238	1.248042354	1.164501355	1.337576561	3.65E-10
AC008105.3	1.05E-05	0.053898891	0.010549385	1.055377889	1.033780506	1.077426477	3.24E-07
AC010491.1	6.18E-06	0.527467098	0.088078403	1.694634525	1.425947636	2.013949251	2.12E-09
AC018648.1	2.58E-08	0.277642344	0.043255186	1.320014002	1.212717534	1.436803638	1.37E-10
AL117379.1	4.60E-06	0.084364776	0.016167744	1.088025707	1.054088676	1.123055361	1.81E-07
Z98200.1	2.36E-05	0.536254533	0.098985861	1.709591637	1.408106384	2.075626954	6.04E-08
AC020907.4	1.06E-05	0.095496896	0.01839181	1.100205407	1.061252284	1.140588299	2.08E-07

AC013731.1	8.54E-05	0.424310293	0.079343374	1.528535814	1.308393617	1.785717772	8.90E-08
AC068722.2	2.94E-05	0.264053036	0.063322827	1.302197258	1.150207748	1.474270802	3.05E-05
AL645940.1	1.03E-05	0.312454051	0.060635026	1.366775138	1.2136248	1.5392519	2.56E-07
AC026401.3	2.49E-06	0.062432673	0.008074599	1.064422793	1.047709917	1.081402269	1.06E-14
LINC01711	1.32E-06	0.060821859	0.007177486	1.062709585	1.047864459	1.077765023	2.37E-17
LINC02352	3.10E-07	0.435087474	0.081576094	1.54509821	1.316795677	1.812983228	9.63E-08
LINC02195	8.69E-06	0.193828964	0.034754691	1.213888646	1.133954433	1.299457547	2.45E-08
AC023043.1	1.57E-07	0.171950091	0.037149885	1.187618559	1.104218289	1.277317949	3.68E-06
ITPR1-DT	6.67E-05	0.092693182	0.023472447	1.097125066	1.047795078	1.1487775	7.85E-05
MELTF-AS1	1.65E-07	0.075243743	0.009801118	1.078146909	1.057633542	1.099058144	1.63E-14
AC048341.2	2.13E-06	0.035494384	0.006701962	1.036131829	1.022610611	1.049831828	1.18E-07
AL139349.1	7.02E-05	0.030691141	0.006712181	1.031166969	1.017690157	1.044822249	4.82E-06
LINC01004	7.57E-05	0.088421251	0.015737115	1.09244822	1.059266908	1.12666893	1.92E-08
AC002398.1	6.04E-06	0.332912859	0.059003555	1.395025729	1.242677115	1.566051841	1.68E-08
LINC00460	3.31E-06	0.092150886	0.012070184	1.096530261	1.070893943	1.122780291	2.27E-14
AC002553.1	7.15E-05	0.098473252	0.024899793	1.103484888	1.050924816	1.158673654	7.66E-05
IGBP1-AS1	7.00E-06	0.281076845	0.059635824	1.324555385	1.17844147	1.488785834	2.44E-06
LINC01507	1.20E-08	−0.079784334	0.013819329	0.923315453	0.89864274	0.948665568	7.77E-09
AC010864.1	1.14E-05	0.472209268	0.082001733	1.603532916	1.365456527	1.883119501	8.49E-09
AC018809.1	2.32E-05	0.368251985	0.082955851	1.445206164	1.228337349	1.700364202	9.03E-06
AL589669.1	2.09E-05	−0.020751385	0.004680204	0.979462443	0.970518886	0.988488418	9.26E-06
AP006545.2	3.98E-05	0.546884916	0.084930784	1.72786219	1.462904201	2.040808787	1.20E-10
AL035413.1	1.75E-07	−0.066156467	0.013003994	0.935984402	0.912430075	0.960146782	3.63E-07
AC103706.1	3.41E-08	0.202888225	0.022953853	1.224935543	1.171048498	1.281302259	9.66E-19
ASB16-AS1	5.46E-05	0.12168514	0.030855102	1.129398444	1.063122413	1.199806183	8.02E-05
AL021707.6	7.77E-05	0.038771777	0.009458693	1.039533211	1.020439156	1.058984546	4.15E-05
AC116407.2	7.24E-05	0.068924137	0.014139443	1.07135493	1.042072311	1.101460401	1.09E-06
AC012645.4	6.58E-06	0.315272933	0.080400704	1.370633351	1.170803714	1.604569374	8.81E-05
AC027796.4	9.20E-05	0.098299658	0.018795261	1.103293347	1.063389688	1.144694389	1.69E-07
MHENCR	3.51E-05	0.019830846	0.004129878	1.020028783	1.011805575	1.028318824	1.57E-06
SNHG3	6.69E-05	0.056756351	0.008006512	1.058397902	1.041918657	1.075137786	1.35E-12
AC092757.3	2.78E-06	0.224063843	0.044451374	1.251150894	1.146760203	1.365044371	4.64E-07
AL021392.1	6.09E-05	0.199257227	0.044761861	1.220495869	1.117982354	1.332409373	8.53E-06
AL358472.3	4.42E-05	0.115429547	0.026660183	1.122355438	1.065214884	1.182561142	1.49E-05
AC010973.2	6.67E-07	0.387708924	0.06563578	1.473600792	1.295718229	1.675903948	3.48E-09
HOTAIRM1	7.06E-07	0.092726452	0.017803251	1.097161568	1.059537743	1.136121403	1.90E-07
AC064836.3	1.57E-05	0.141773142	0.026058663	1.152315207	1.094939489	1.212697459	5.31E-08
AL121782.1	5.31E-06	0.401472324	0.068288757	1.494022764	1.306861968	1.707987587	4.13E-09
AC027373.1	1.55E-07	0.326770721	0.067972603	1.386483549	1.213546261	1.584065391	1.53E-06
SNHG17	3.94E-07	0.132553085	0.021821792	1.141739623	1.093937047	1.191631065	1.24E-09
AL035071.1	3.21E-06	0.048296868	0.010327677	1.049482167	1.028452241	1.070942116	2.92E-06
AC005586.1	7.35E-06	0.077086034	0.014336941	1.080135001	1.050205801	1.110917135	7.58E-08
AC156455.1	9.80E-05	0.048241692	0.008564606	1.049424262	1.03195532	1.067188918	1.77E-08
AC025171.4	7.85E-07	0.083842226	0.01724214	1.087457308	1.051321824	1.124834821	1.16E-06
AC008870.2	3.59E-06	0.226716622	0.041953878	1.254474327	1.155448444	1.361987068	6.52E-08
DLGAP1-AS2	3.97E-09	0.182129981	0.019116793	1.199770132	1.155648612	1.245576167	1.62E-21
EPB41L4A-DT	6.83E-10	−0.160609112	0.0243253	0.851624896	0.811974922	0.893211039	4.04E-11
AC002070.1	1.31E-06	−0.198186226	0.035681387	0.820217093	0.764815646	0.879631691	2.79E-08
AP000525.1	4.35E-05	0.142501009	0.023924273	1.153154245	1.100330177	1.208514263	2.58E-09
NALT1	9.12E-05	0.133207099	0.028140911	1.142486582	1.081178801	1.207270795	2.21E-06
AC063919.1	3.78E-06	−0.349475019	0.082976973	0.705058135	0.599231728	0.829573853	2.53E-05
Z84485.1	2.40E-05	0.216111139	0.039012497	1.241240322	1.149869031	1.33987219	3.03E-08
AL162377.1	5.59E-06	−0.577522832	0.107536573	0.561287048	0.454621242	0.692979389	7.85E-08
LINC01132	8.60E-05	−0.529654656	0.112342805	0.588808276	0.472440953	0.733838132	2.42E-06
MCCC1-AS1	3.89E-05	0.18202817	0.040714941	1.199647988	1.107636374	1.299303029	7.79E-06
AC080129.2	3.13E-06	0.581579037	0.094039263	1.788860879	1.487750829	2.150913433	6.23E-10

SNHG4	8.25E-07	0.261468229	0.035446618	1.298835676	1.211663411	1.392279488	1.63E-13
U91328.1	5.96E-05	−0.174572934	0.039619747	0.839815601	0.777068937	0.907628924	1.05E-05
ZKSCAN2-DT	8.11E-05	0.145592751	0.030783279	1.156725017	1.088998681	1.228663347	2.25E-06
CAHM	9.09E-05	0.195207797	0.03690409	1.215563551	1.130745455	1.306743918	1.23E-07
AC087741.1	7.40E-05	0.159159837	0.037678445	1.172525345	1.089056192	1.262391871	2.40E-05
AC007938.3	1.14E-07	0.352583554	0.067640889	1.422738526	1.246089001	1.624430447	1.86E-07
AC015912.3	9.13E-09	0.152307839	0.022819704	1.164518665	1.113582217	1.217785001	2.48E-11
AC011005.4	5.54E-05	0.155041758	0.037665168	1.167706721	1.084608816	1.257171217	3.85E-05
U47924.3	1.22E-07	0.261372731	0.038892188	1.298711646	1.203393454	1.401579785	1.81E-11
LINC01605	2.42E-06	0.138603255	0.018177388	1.148668282	1.10846506	1.190329645	2.44E-14
AC020558.2	9.84E-05	0.163766989	0.03807404	1.17793981	1.093237242	1.269205021	1.70E-05
CAPN10-DT	3.08E-05	0.231670537	0.049782649	1.260704305	1.143505265	1.389915197	3.26E-06
AC108673.3	2.24E-05	0.066307305	0.011707291	1.068555039	1.04431528	1.093357431	1.48E-08
AC084876.1	1.57E-07	0.512354035	0.074423507	1.669215966	1.442657129	1.931354226	5.81E-12
AL355388.1	1.09E-07	0.365463538	0.053138466	1.441181896	1.298635449	1.599375142	6.09E-12
AC092143.3	3.15E-05	0.392664019	0.074844243	1.480920744	1.278863762	1.714902177	1.55E-07

Supplementary Table 5. Comparison of gene models published based on time-dependent ROC.

Models	1 years	3 years	5 years	*P*-value	PMID
Our model	0.791	0.783	0.797	$P < 0.001$	−
Yufu Wang et al. [15]	0.715	0.693	0.732	$P < 0.001$	9286942
Ding Wu et al. [16]	0.695	0.678	0.674	$P < 0.001$	9448526
Qianwei Xing et al. [17]	0.724	0.716	0.714	$P < 0.001$	9162755
Yijun Le et al. [18]	0.745	0.762	0.737	$P < 0.001$	10126632
Lei Zhang et al. [19]	0.773	0.698	0.747	$P < 0.001$	10433381
Jiyue Wu et al. [20]	0.734	0.720	0.757	$P < 0.001$	8237220
